# Target specificity, *in vivo* pharmacokinetics, and efficacy of the putative STAT3 inhibitor LY5 in osteosarcoma, Ewing's sarcoma, and rhabdomyosarcoma

**DOI:** 10.1371/journal.pone.0181885

**Published:** 2017-07-27

**Authors:** Peter Y. Yu, Heather L. Gardner, Ryan Roberts, Hakan Cam, Seethalakshmi Hariharan, Ling Ren, Amy K. LeBlanc, Hui Xiao, Jiayuh Lin, Denis C. Guttridge, Xiaokui Mo, Chad E. Bennett, Christopher C. Coss, Yonghua Ling, Mitch A. Phelps, Peter Houghton, Cheryl A. London

**Affiliations:** 1 Medical Student Research Program, The Ohio State University College of Medicine, The Ohio State University Wexner Medical Center, Columbus, Ohio, United States of America; 2 Department of Veterinary Biosciences and Clinical Sciences, The Ohio State University, Columbus, Ohio, United States of America; 3 Center for Childhood Cancer, Nationwide Children’s Hospital, Columbus, Ohio, United States of America; 4 Greehey Children’s Cancer Research Institute, University of Texas Health Science Center, San Antonio, Texas, United States of America; 5 Comparative Oncology Program, Center for Cancer Research, National Cancer Institute, National Institutes of Health, Bethesda, Maryland, United States of America; 6 Arthur G. James Comprehensive Cancer Center, The Ohio State University Wexner Medical Center, Columbus, Ohio, United States of America; 7 Department of Cancer Biology and Genetics, The Ohio State University Wexner Medical Center, Columbus, Ohio, United States of America; 8 Center for Biostatistics, The Ohio State University Wexner Medical Center, Columbus, Ohio, United States of America; 9 Medicinal Chemistry Shared Resource, The Ohio State University Wexner Medical Center, Columbus, Ohio, United States of America; 10 Pharmacoanalytic Shared Resource, The Ohio State University Wexner Medical Center, Columbus, Ohio, United States of America; 11 Cummings School of Veterinary Medicine, Tufts University, Grafton, Massachusetts, United States of America; Universite de Nantes, FRANCE

## Abstract

**Background:**

STAT3 is a transcription factor involved in cytokine and receptor kinase signal transduction that is aberrantly activated in a variety of sarcomas, promoting metastasis and chemotherapy resistance. The purpose of this work was to develop and test a novel putative STAT3 inhibitor, LY5.

**Methods and findings:**

An *in silico* fragment-based drug design strategy was used to create LY5, a small molecule inhibitor that blocks the STAT3 SH2 domain phosphotyrosine binding site, inhibiting homodimerization. LY5 was evaluated *in vitro* demonstrating good biologic activity against rhabdomyosarcoma, osteosarcoma and Ewing’s sarcoma cell lines at high nanomolar/low micromolar concentrations, as well as specific inhibition of STAT3 phosphorylation without effects on other STAT3 family members. LY5 exhibited excellent oral bioavailability in both mice and healthy dogs, and drug absorption was enhanced in the fasted state with tolerable dosing in mice at 40 mg/kg BID. However, RNAi-mediated knockdown of STAT3 did not phenocopy the biologic effects of LY5 in sarcoma cell lines. Moreover, concentrations needed to inhibit *ex vivo* metastasis growth using the PuMA assay were significantly higher than those needed to inhibit STAT3 phosphorylation *in vitro*. Lastly, LY5 treatment did not inhibit the growth of sarcoma xenografts or prevent pulmonary metastasis in mice.

**Conclusions:**

LY5 is a novel small molecule inhibitor that effectively inhibits STAT3 phosphorylation and cell proliferation at nanomolar concentrations. LY5 demonstrates good oral bioavailability in mice and dogs. However LY5 did not decrease tumor growth in xenograft mouse models and STAT3 knockdown did not induce concordant biologic effects. These data suggest that the anti-cancer effects of LY5 identified *in vitro* were not mediated through STAT3 inhibition.

## Introduction

Despite marked improvements in outcome associated with dose-intensive chemotherapy, advanced or metastatic sarcoma remains a clinical challenge. Approximately 30–40% of children with osteosarcoma (OS) still die due to metastatic disease, and survival rates for children with metastatic rhabdomyosarcoma (RMS) and Ewing’s sarcoma (ES) are similarly dismal and have not improved in over 15 years [1–3]. Signal transducer and activator of transcription 3 (STAT3) is a transcription factor with roles in cell growth and survival that is aberrantly activated in both childhood and adult solid tumors, OS, RMS and ES [4–7]. Dysregulation of STAT3 has been associated with increased tumor cell proliferation, survival, invasion and immunosuppression *in vitro* [8], and mounting evidence supports the critical role of STAT3 as an oncogene [9–10].

The predominant oncogenic role of STAT3 in many solid tumors has generated interest in exploring STAT3 as a relevant target for therapeutic intervention. However, finding a strategy for specific and effective STAT3 inhibition has proven challenging [11]. STAT3 contains an N-terminus dimerization domain, a Src homology 2 (SH2) domain, and a central DNA binding domain [12]. Over a dozen synthetic STAT3 inhibitors that directly target STAT3 have been explored and are currently undergoing clinical trials, but none have been approved for clinical use [13,14].

We previously investigated the allosteric small molecule STAT3 inhibitor, LLL12, demonstrating that it inhibits proliferation and induces apoptosis in childhood sarcoma cell lines [15,16], although this drug had poor pharmacokinetic properties. Using an *in silico* site-directed fragment-based drug design, we developed a derivative of LLL12, 5,8-dioxo-6-(pyridin-3-ylamino)-5,8-dihydronaphthalene-1-sulfonamide (LY5) [17]. LY5 has greater predicted oral bioavailability and binding activity to the STAT3 SH2 domain compared to its predecessor LLL12, and was designed to inhibit both STAT3 activation and dimerization by blocking the SH2 domain phosphotyrosine binding site [17]. LY5 has shown activity in *in vivo* models of breast and colon cancer as well as *in vitro* models of medulloblastoma alone and in combination with chemotherapy or radiation [17–19]. STAT3 inhibition using LY5 has also been explored for treatment of drug-resistant cancer cells. The combination of LY5 with a MEK inhibitor (trametinib) for KRAS-mutated pancreatic and colon cancer cells showed anti-tumor cell efficacy *in vitro* [20]. In RMS, the combination of LY5 with doxorubicin, cisplatin, and a MEK inhibitor (Selumitinib, AZD6244) showed stronger inhibitory effects compared to single-agent therapy alone [21].

The purpose of this body of work was to characterize the ability of LY5 to inhibit STAT3 phosphorylation in a variety of sarcoma cell lines *in vitro*, and evaluate the pharmacokinetic (PK) and anti-tumor properties of LY5 *in vivo*. Our data showed that LY5 was efficacious *in vitro* in inhibiting phosphorylation of STAT3 in RMS, OS, and ES cell lines at micromolar concentrations, but knockdown of STAT3 did not confer resistance to LY5. While LY5 demonstrated good PK parameters and oral bioavailability *in vivo*, inhibition of the tumor growth of patient-derived xenograft (PDX) models or metastatic disease in lung metastasis models was not observed. These data suggest that while LY5 is capable of inhibiting STAT3 phosphorylation, the anti-cancer effects of LY5 are likely due to as yet undefined off-target effects.

## Materials and methods

### Cell lines and reagents

The human RMS cell lines (RD, and RD2) and human OS cell line SJSA were purchased from American Type Cell Culture Collection (ATCC, Manassas, VA). The human OS cell line (OS-17), human OS PDX model (OS-1), human RMS cell line (RH30), human RMS PDX models (RH10 and RH36), and human ES cell lines (EW8, ES-3, ES-6, ES-7, and ES-8) were generously provided by the Pediatric Preclinical Testing Program (PPTP). The human RMS JR-1 cell line was established at St. Jude Children’s Research Hospital, and characteristics have been described [22]. Human OS cell line MG63.3 and murine OS cell line K7M2 were generated in Dr. Chand Khanna’s laboratory [23]. All cell lines were cultured in complete media (DMEM or RPMI) containing 10% fetal bovine serum, 4 mM L-glutamine, sodium pyruvate and 1% penicillin/streptomycin. Cell lines were maintained in a humidified 37° C incubator with 5% CO_2_.

An *in silico* site-directed fragment-based drug design was used to develop a novel compound, LY5, synthesized by Chenglong Li’s Laboratory (College of Pharmacy, The Ohio State University) and purified as previously described [17]. For *in vitro* treatment of sarcoma cell lines, LY5 powder was dissolved in sterile dimethylsulfoxide (DMSO) and the stock solution was stored at -20° C. The LY5 dosing solution for *in vivo* administration in mice consisted of DMSO/Solutol/20% hydroxypropyl beta-cyclodextrin (v/v/v: 5/10/85). The dosing solution was freshly prepared immediately prior to the first dose and pipetted to mix prior to each injection. A 1 mg/ml dosing solution of LY5 was prepared in a 5% DMSO/30% hydroxypropyl beta-cyclodextrin formulation and sterile-filtered for dosing of normal beagle dogs. Lastly, capsules containing 25 or 50 mg LY5 powder were prepared for oral administration to normal hound dogs.

### Cell lysis, Western blotting, and cytokine stimulation

Cell lysis, protein isolation and immunoblotting were performed as described previously [24]. Twenty micrograms of protein were resolved using 4–12% Bis-Tris polyacrylamide gels. Gels were transferred to PVDF membranes and subjected to blocking and incubation with primary and secondary antibodies. The primary antibodies used in this study included: phospho-STAT3 (Tyr705, Cell Signaling, Boston, MA and Abcam, Cambridge, MA), total STAT3 (Cell Signaling and Abcam), phospho-STAT1 (Cell Signaling), total STAT1 (Cell Signaling), phospho-STAT2 (Cell Signaling), phospho-STAT4 (Cell Signaling), phospho-STAT6 (Cell Signaling), and GAPDH (Cell Signaling). Protein bands were detected using Western Lightning ® Plus Enhanced Chemiluminescence substrate (Perkin Elmer, Waltham, MA) and HyBlot CL ® Autoradiography film (Denville Scientific, Holliston, MA). The cytokines used in this study included: interferon gamma (IFN-γ, Cell Signaling), interferon alpha (IFN-α, Cell Signaling), interleukin-4 (IL-4, Cell Signaling), interleukin-6 (IL-6, Cell Signaling), and oncostatin M (OSM, Cell Signaling).

### Cell viability assay

Sarcoma cell lines were seeded in 24-well plates in 1X RPMI medium containing 10% fetal bovine serum. Cells were then incubated with DMSO (control) or concentrations of LY5 ranging from 10 nM to 10 μM for 96 hrs. At the end of the incubation period, Alamar Blue (Invitrogen, Carlsbad, CA) was added to cells at one-tenth the volume of the cell culture medium and incubated for 3 hrs at 37° C in the dark. Fluorescence was then measured at an excitation/emissions wavelength of 570/590 nm and emission wavelength of 590 nm.

### siRNA transfection

Cell lines were transfected with 10 nM of AllStars negative control siRNA or human STAT3-targeting siRNA #7 (5’-CAGCCTCTCTGCAGAATTCAA-3’) (Qiagen, Germantown, MD) using Lipofectamine RNAiMAX (Life Technologies, Carlsbad, CA) according to the manufacturer’s instructions. After 72 hrs of transfection, cells were harvested and lysed for immunoblotting. For the combined STAT3 knockdown–LY5 cell viability assay, negative control siRNA or STAT3 siRNA #7 was transfected into cell lines. After 48 hrs, cells were treated with 0.5 μM or 1 μM of LY5 for another 48 hrs. Cell viability was then analyzed using Alamar Blue as described above.

### Assessment of plasma PK profiles of LY5 in mice

Single-dose toxicity studies in mice were performed to identify the maximum tolerated dose of LY5. Additionally, 30 ICR mice (Harlan Laboratories, Indianapolis, IN) were divided into 3 groups to be dosed with 5 mg/kg LY5 (2 mg/ml) by oral (PO), intraperitoneal (IP) or intravenous (IV) routes to establish oral bioavailability. Animals were euthanized and blood was collected at allocated time points (5, 10, 20, 30 and 60 minutes, 2, 4, 6, 8 and 24 hrs) after LY5 administration, plasma was separated by centrifugation and frozen immediately on dry ice. Samples were stored at -80° C prior to analysis.

### Assessment of PK and clinical toxicities of LY5 in normal dogs

LY5 was evaluated in two normal purpose-bred beagle dogs (female 8.8 kg; male 11.2 kg) receiving a single dose of 1mg/kg LY5 IV then orally by gavage 7 days later to establish the PK profile and assess the spectrum of clinical toxicities associated with LY5 administration. The oral dosing was repeated in the fed and fasted state. The starting dose was determined based on allometric scaling from the maximal tolerated dose studies performed in mice. Dogs were washed out for 30 days, and then given a dose of 25 mg in a capsule orally, followed by a 50 mg capsule dose 14 days later. Full PK sampling was performed over 24 hours following each dose of LY5. The dogs received LY5 and were used for the PK analysis but were not euthanized upon conclusion of the study.

### PK analysis

The liquid chromatography-mass spectrometry system utilized for these analyses consisted of a Dionex Ultimate 3000 RSLCnano High Performance Liquid Chromatography system and a Thermo Finnigan Quantum Ultras EMR mass spectrometer with an electrospray ionization source. Positive ionization mode was used for both LY5 and the internal standard, hesperetin. Plasma samples were collected and analyzed by the OSU James Comprehensive Cancer Center Pharmacoanalytical Shared Resource. Pharsight Phoenix WinNonlin software using non-compartmental analysis was used to generate PK parameters including maximum plasma concentration (Cmax), amount of time that drug is present at Cmax (Tmax), and total area under the curve (AUC). AUC all and AUC last were calculated by linear trapezoidal method of concentration-time curve up to the last measured point. AUC inf-obs was calculated by log-linear extrapolation of the terminal phase beyond the last collection time. Bioavailability (F) was calculated for IP and PO administration routes. Plasma stability and liver microsomal stability of LY5 were also assessed through this shared resource.

### Evaluation of pulmonary metastasis *in vivo*

The ability of LY5 to inhibit pulmonary metastases was evaluated using a murine model of OS pulmonary metastasis. 8-week-old C.B-17 *scid* -/- female mice (Envigo, Madison, WI) received IV inoculation with 10^6^ OS-17-luc cells expressing a luciferase reporter gene. OS-17-luc cells were generated by infecting OS-17 wild type cells with lentivirus containing the firefly luciferase under a CMV promoter (Cellomics Technology, Hallethorpe, MD) and selected with 0.5 ug/mL puromycin (Thermo Fisher Scientific, Waltham, MA). Beginning 24 hrs after inoculation, mice received vehicle (n = 10) or 20 mg/kg LY5 once daily via oral gavage (n = 9, one mouse died within 24 hrs after cell inoculation). LY5 was prepared by diluting a 40 mg/mL solution/slurry with a 50% propylene glycol/1% Tween 20 in water vehicle immediately before administration. Mice were treated with LY5 for 30 days, and bioluminescent imaging (after IP luciferin administration) was performed to determine pulmonary metastatic disease load. To investigate the pharmacodynamics (PD) effects of LY5, pSTAT3 (p-Tyr705, ab76315, Abcam) and total STAT3 (ab119352, Abcam) were evaluated in pulmonary metastatic lesions using IHC in lung samples from mice euthanized 24 hrs after their last dose.

### *Ex vivo* pulmonary metastasis assay (PuMA)

Murine K7M2 and human MG63.3 OS cells were treated with increasing concentrations of LY5 for 2 hrs or 7 hrs. Western blotting was performed for pSTAT3 and STAT3 as indicated above to confirm inhibition of STAT3 phosphorylation in the cell lines prior to initiation of the PuMA assay. In addition, a sulforhodamine B colorimetric assay was performed to assess the cytotoxicity of LY5 in K7M2 and MG63.3 cells as previously described [25].

GFP-positive K7M2 and MG63.3 cells (3 x 10^5^ MG63.3; 5 x 10^5^ K7M2) were administered via tail vein injection into mice. Following humane euthanasia, the lungs were insufflated with a culture medium/agarose mixture to a constant hydrostatic pressure of 20 cm H_2_O and prepared for *ex vivo* culture as previously described [26]. Lung sections were cultured in complete media containing LY5 (0, 0.1, 1, 10 or 50 μM) on sterile Gelfoam using 6 lung sections per treatment condition. Media was changed every 48 hrs and lung slices were imaged with a fluorescent microscope every 7 days for a total of 21 days.

### *In vivo* tumor xenograft study

C.B-17SC *scid*^*-/-*^ female mice (Taconic Farms, Germantown NY), were used to propagate subcutaneously implanted tumors. All mice were maintained under barrier conditions and experiments were conducted using protocols and conditions approved by the institutional animal care and use committee at University of Texas Health Science Center at San Antonio. Ten mice were used in each control or treatment group. Tumor volumes (cm^3^) were determined and responses were determined using three activity measures as previously described [27].

### Statistical methods

The data were analyzed by using GraphPad Prism software (GraphPad Software, Inc., San Diego, CA). Data are presented as mean ± standard deviation. Data were analyzed by one-way analysis of variance (ANOVA), and Tukey’s post-test was used to assess differences between groups. Differences were considered statistically significant at p < 0.05. For *in vivo* xenograft models, an event was defined as four times the tumor volume at baseline similar to the PPTP criteria [27]. The probabilities of time-to-event among groups were compared using log-rank test. The anti-tumor efficacy of LY5 was analyzed by using the statistical pipeline developed in PPTP [27].

### Ethics statement

Murine and canine experiments were conducted in accordance with the institutional animal care and use committee of the Research Institute at Nationwide Children’s Hospital and the Ohio State University approved protocols (IACUC# 2009A0196-R1) and University of Texas Health Science Center at San Antonio (IACUC# 15015x) for patient-derived xenograft models, designed to minimize the numbers of mice used and to minimize any pain or distress. The institutional review boards/ethics committees at the respective institutions specifically approved this study.

## Results

### LY5 selectively inhibited cytokine-driven STAT3 phosphorylation *in vitro*.

**Fig 1:**
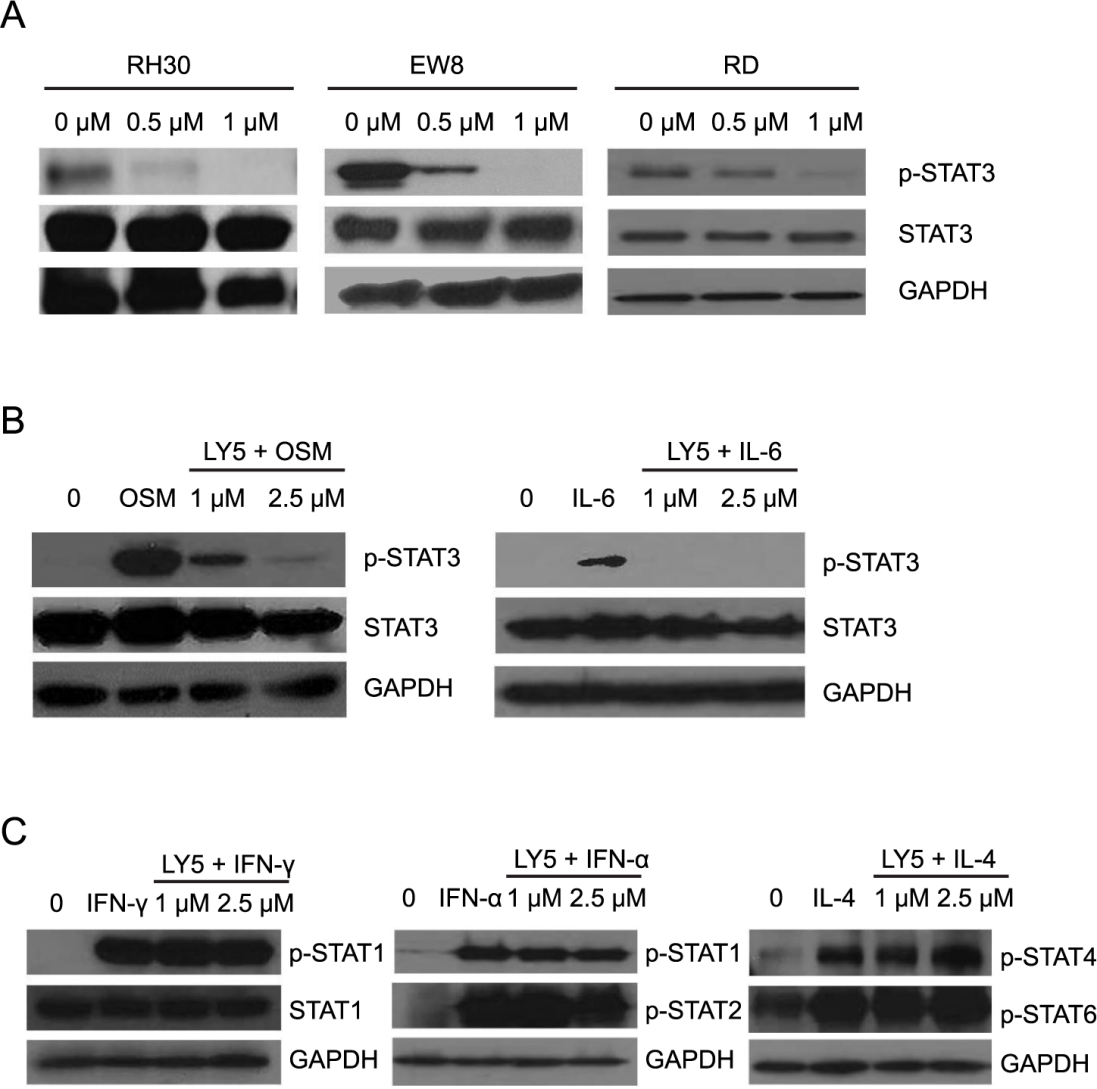
LY5 inhibits pSTAT3 in sarcoma cell lines. (A) The tumor cell lines RH30, EW8, and RD were treated with increasing concentrations of LY5 for 30 minutes. Cell lysates were collected for Western blotting of p-STAT3, total STAT3, and GAPDH. GAPDH was used as loading control. (B-C) The SJSA cell line was serum-starved overnight and then left untreated or treated with LY5 for two hours followed by stimulation with 50 ng/mL of OSM, IL-6, IFN-γ, IFN-α, or IL-4 for 30 minutes prior to collection of cell lysates for Western blotting of p-STAT3, total STAT3 p-STAT1, total STAT1, p-STAT2, p-STAT4, or p-STAT6. GAPDH was used as loading control.

LY5 was previously demonstrated to inhibit STAT3 phosphorylation in RMS cells [21]. Consistent with these results, we found that LY5 inhibited basal STAT3 phosphorylation at Tyr705 in ES and OS cells in addition to RMS cells (Fig 1A) [28,29]. Furthermore, LY5 effectively blocked IL-6 and OSM induced STAT3 phosphorylation in the SJSA OS cell line, with OSM providing a stronger induction of pSTAT3 (Fig 1B). To determine the specificity of STAT blockade, we asked whether LY5 affected the phosphorylation of other family members including STAT1, STAT2, STAT4, and STAT6 [30–32]. In cell line SJSA, LY5 did not inhibit phosphorylation of STAT1, STAT2, STAT4 or STAT6 induced by IFN-γ, IFN-α, or IL-4 (Fig 1C). To further define any potential off-target effects, we profiled the direct (steric) or indirect (allosteric) binding of LY5 to the binding sites of 96 human protein or lipid kinases (*KINOMESCAN*, www.kinomescan.com). LY5 exhibited no binding to any of the kinases at 1 μM and binding only to polo-like kinase 3 (PLK3) at 5 μM (S1 Fig), supporting the target specificity of drug.

### LY5 decreased *in vitro* viability of RMS, ES and OS cells

**Fig 2:**
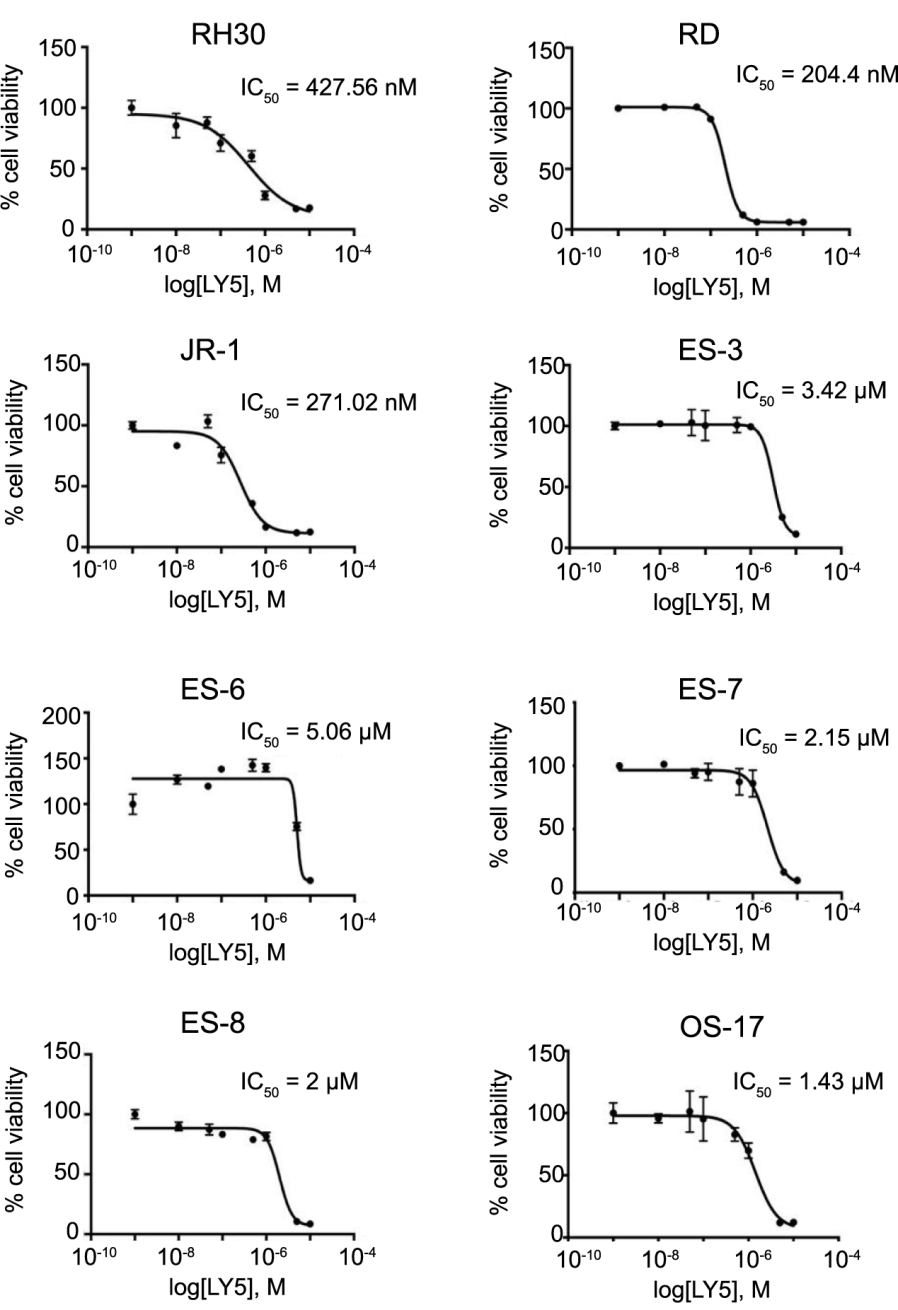
Biologic activity of LY5 in sarcoma cell lines. Dose response curves for LY5: RMS cell lines (RH30, RD, JR-1), ES cell lines (ES-3, ES-6, ES-7, ES-8), and an OS cell line (OS-17) were treated with LY5 (concentrations ranging from 10 nM to 10 μM) for 96 hours. Cell viability was determined using Alamar Blue staining. IC_50_ values are indicated for each cell line. Analyses were performed in triplicate and data are plotted as means ± standard deviation.

To determine the relevance of decreased STAT3 phosphorylation in sarcoma cells, we analyzed *in vitro* cell viability of RMS, ES, and OS cells after treatment with LY5 (Fig 2). LY5 decreased the viability of sarcoma cell lines with IC_50_ concentrations in a nanomolar range. The human rhabdomyosarcoma cell lines RD, JR-1, and RH30 demonstrated the highest sensitivity to LY5 exposure (IC_50_ values 0.2–0.4 μM). ES and OS cells were also sensitive to LY5 administration (IC_50_ values 1.4–5.1 μM). These data supported our further study of LY5 *in vivo*.

### LY5 demonstrated favorable (PK) in mice and dogs

**Fig 3:**
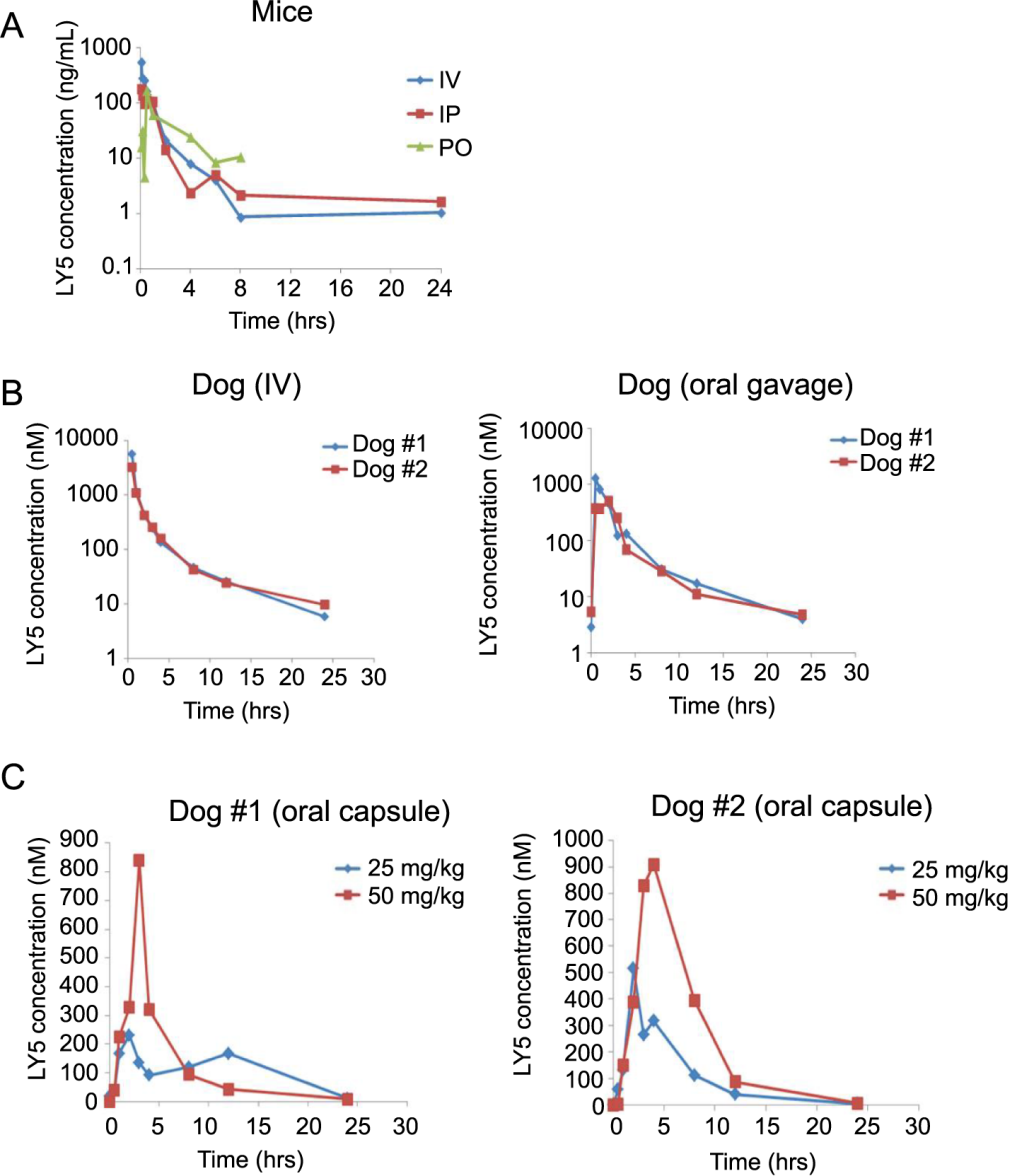
Pharmacokinetic analysis of LY5 in mice and normal dogs. (A) Semi logarithmic plot of LY5 plasma concentration over time in mice administered 5 mg/kg LY5 IV, IP, or PO (10 mice of each, n = 30 total). Animals were euthanized at various time points between 5 minutes and 24 hours to collect plasma samples. (B) Semi logarithmic plot of LY5 plasma concentrations in normal dogs administered a single 1 mg/kg dose of LY5 IV or through oral gavage. (C) Linear plot of concentration-time profile of LY5 in normal fasted dogs administered as powder in an oral capsule (25 and 50 mg/kg).

We used both murine and canine models to evaluate the PK profile of LY5. In a pilot study to gain an initial understanding of plasma PK, mice were dosed with 5 mg/kg LY5 via IV, IP, or PO routes (10 mice each, 30 mice total), and animals were euthanized at various time points between 5 minutes and 24 hours to collect plasma for analytical analysis (Fig 3A). LY5 showed 69.5% bioavailability for IP administration and 78.6% for PO administration (S1A Table). The 24 hr time point for the PO route was not collected, but the PO route had higher LY5 concentration than the IP and IV routes at 4, 6, and 8 hrs. For PO administration, LY5 plasma concentration was below the limit of quantification at time points of 2 hrs and 24 hrs. No toxicity was noted at any of the doses received via IV, oral, or IP routes. Approximately 90% of drug was protein-bound in mice.

We subsequently examined systemic bioavailability of LY5 in normal beagle dogs with IV bolus and oral gavage administration at a dose of 1 mg/kg. The administration of 1 mg/kg LY5 in normal dogs was well tolerated without apparent acute clinical toxicities during the 24 hour PK collection timeframe. The overlaid plasma concentration and time profiles of LY5 with IV bolus administration are shown in Fig 3B. The first oral PK experiment in beagle dogs was performed under fed conditions. Dog #2 vomited 10 minutes after drug administration and plasma drug levels were correspondingly lower in that dog. The second oral PK experiment was performed one week later under fasted conditions and no vomiting occurred (Fig 3B). LY5 was found to be orally bioavailable, with 44 and 52% of drug entering systemic circulation for the fasted female and male dogs, respectively. Oral bioavailability was increased from 14 to 52% under fasting versus fed conditions, respectively (S1B Table). The lower oral bioavailability in dogs compared to mice in combination with the increased stability of LY5 when incubated with canine liver microsomes (data not shown), suggests that there is either lower oral absorption in the dog or an unidentified extrahepatic clearance mechanism. The same two normal beagle dogs were dosed with oral capsules filled with LY5 powder (25mg and 50 mg) under fasted conditions (Fig 3C). The AUC was similar for administration of oral capsule as compared to oral gavage of 1 mg/kg LY5 solution under fasted conditions, and administration of LY5 capsules resulted in dose-proportional pharmacokinetics (S1C Table). In a pilot study to gain understanding of chronic dosing of mice, once daily gavage administration of LY5 at 18 mg/kg and 36 mg/kg over 14 days resulted in higher plasma concentrations from 30 min post dose and up to 6 hours post dose (data not shown). These data demonstrate that biologically achievable exposures of LY5 in mice and dogs are consistent with drug concentrations needed to inhibit STAT3 phosphorylation.

### LY5 did not inhibit *in vivo* lung metastasis formation

**Fig 4:**
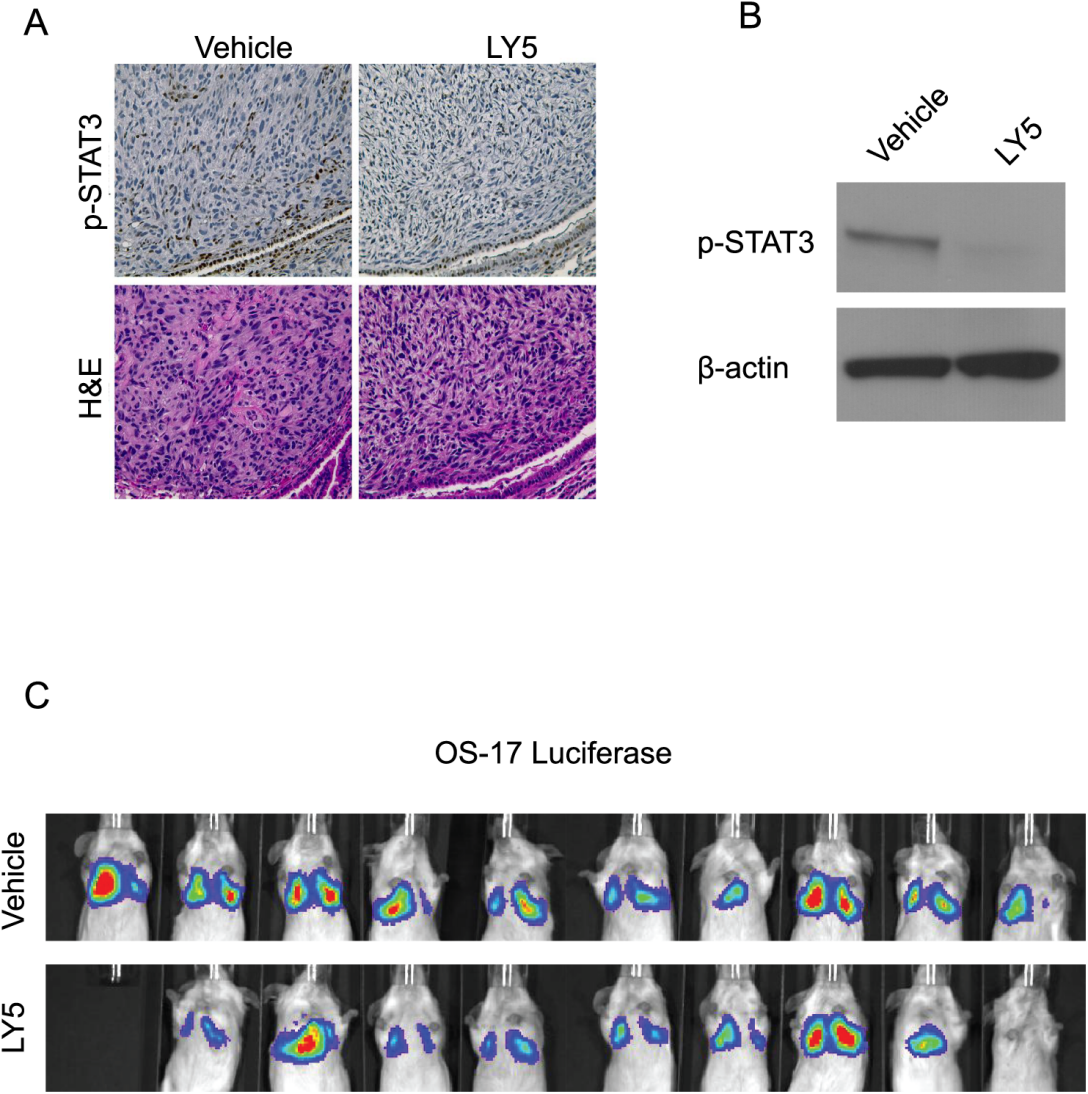
LY5 inhibits STAT3 phosphorylation in lung metastases but does not inhibit lung metastasis formation. (A-B) IHC staining of lung metastasis from mice treated with LY5. The metastatic tumor sections were stained with pSTAT3 or hematoxylin and eosin. Western blotting was performed to confirm inhibition of STAT3 phosphorylation in lung tumor sections. (C) Mice were administered OS-17 cells stably expressing luciferase by tail vein injection and subsequently treated with LY5. After 30 days, imaging was performed to evaluate the effects of LY5 on the development of pulmonary metastasis.

The biologic activity of LY5 was evaluated using an *in vivo* murine model of metastatic OS. Mice received 1 x 10^6^ OS-17 cells stably expressing luciferase by tail vein injection and were then treated with 20 mg/kg LY5 daily for 30 days. Consistent with the *in vitro* study, LY5 inhibited STAT3 phosphorylation in pulmonary metastatic lesions as demonstrated by immunohistochemistry (Fig 4A) and Western blotting (Fig 4B). However, no difference in the development of pulmonary metastatic lesions was observed in mice receiving vehicle versus LY5 based on luciferase expression (Fig 4C). These results indicated that LY5 lacked anti-tumor efficacy *in vivo* despite PD analysis demonstrating inhibition of STAT3 phosphorylation in the presence of LY5.

### Inhibition of STAT3 phosphorylation by LY5 was disconnected from *ex vivo* OS growth

**Fig 5:**
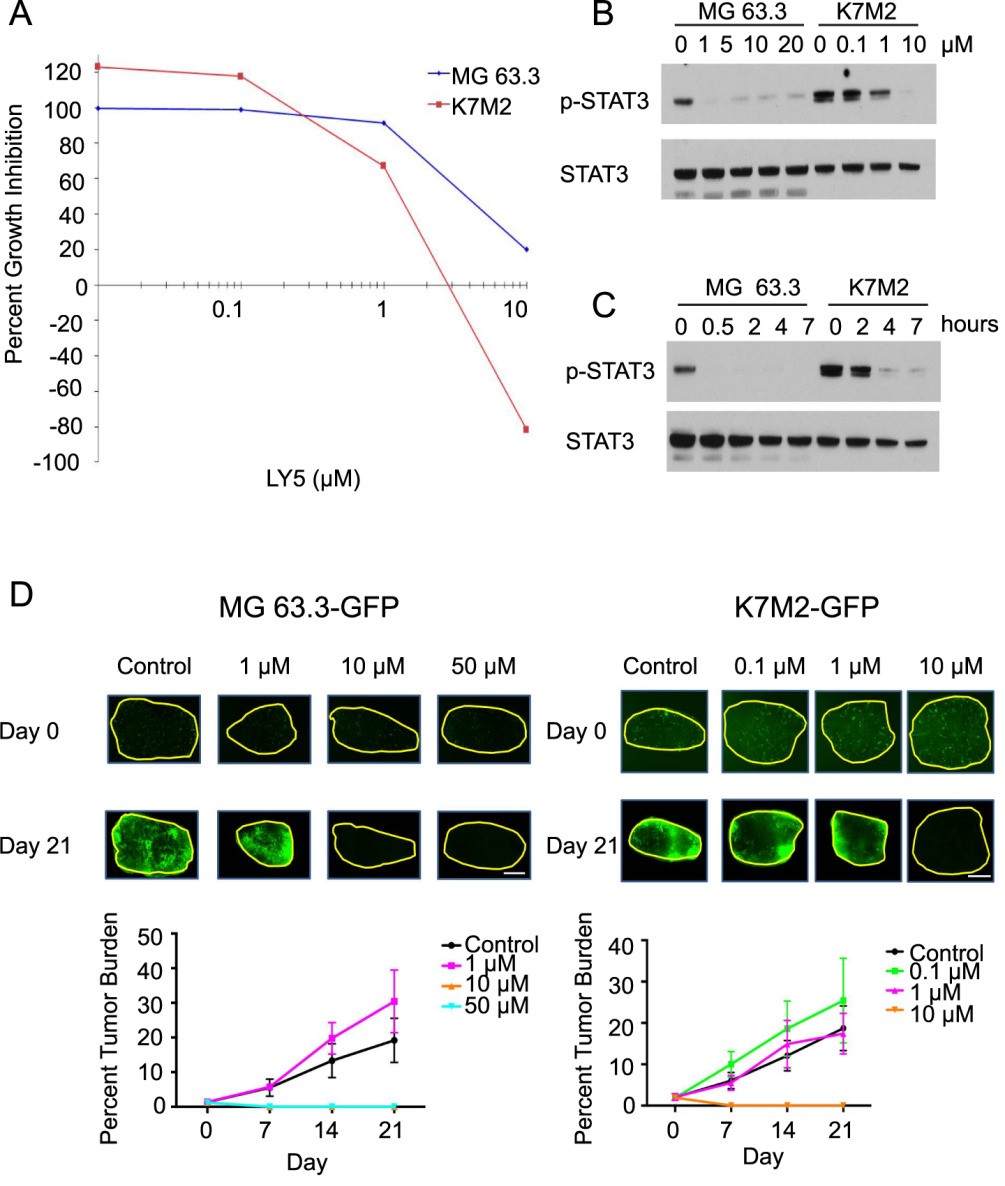
LY5 does not inhibit growth of *ex vivo* pulmonary metastasis. MG63.3 and K7M2 cell lines were exposed to increasing concentrations of LY5 to determine the IC_50_ (A). Western blot analysis was performed to determine the dose and duration of LY5 exposure required to inhibit STAT3 phosphorylation (B-C). Serial imaging of fluorescently labeled MG63.3 and K7M2 cells was performed in *ex vivo* LY5 treated lung sections (D). Metastatic burden was quantified by evaluation of the percent tumor burden (mean normalized fluorescent area). Data are reported as the mean ± standard deviation. In MG 63.3 experiment, p < 0.0001 Kruskal-Wallis test, p < 0.05 10 μM vs control at days 7, 14, and 21, 10 μM vs 1 μM at days 7, 14, and 21, 50 μM vs control at days 7, 14, and 21, and 50 μM vs 10 μM at days 7, 14, and 21 post-test Dunn. In K7M2 experiment, p < 0.0001 Kruskal-Wallis test, p < 0.05 10 μM vs control at days 14 and 21, 10 μM vs 0.1 μM at days 14 and 21, and 10 μM vs 1 μM at days 14 and 21, post-test Dunn.

Given that LY5 exhibited *in vitro* cell viability effects but no *in vivo* efficacy against OS lung metastasis growth, we evaluated the biologic activity and target specificity of LY5 in 2 additional OS cell lines (MG63.3 and K7M2) using Western blotting and an *ex vivo* pulmonary metastasis assay (PuMA) [26]. Both lines were sensitive to LY5 *in vitro* as determined by cell viability assays (Fig 5A; IC_50_ values 3 and 1.5 μM, respectively). Upon treatment of LY5 at various doses for 2 hrs, LY5 decreased STAT3 phosphorylation at a concentration of 1 μM in both cell lines (Fig 5B). STAT3 phosphorylation was effectively inhibited with exposure to 1 μM LY5 for 0.5 hrs in the MG 63.3 cell line and for 4 hrs in the K7M2 cell lines (Fig 5C). Using the established *ex vivo* PuMA assay to test efficacy of LY5 at various concentrations, we found that concentrations of 10 and 50 μM LY5 were required to inhibit growth of MG 63.3 metastatic lesions in this assay (Fig 5D). Higher concentrations of LY5 (10 μM and 50 μM) significantly decreased percent tumor burden of MG 63.3 cells. Similarly, K7M2 cells showed complete inhibition of STAT3 *in vitro* only after 10 μM LY5 (Fig 5B), which was reflected in the PuMA assay with a reduction at 10 μM only (Fig 5D). Therefore, the concentrations of LY5 necessary to inhibit tumor growth in the PuMA assay were discordant with those required for inhibition of STAT3 phosphorylation, suggesting that the biologic effects of LY5 were disconnected from its effects on the activity of STAT 3.

### LY5 exhibited off-target effects that decreased *in vitro* cell viability

**Fig 6:**
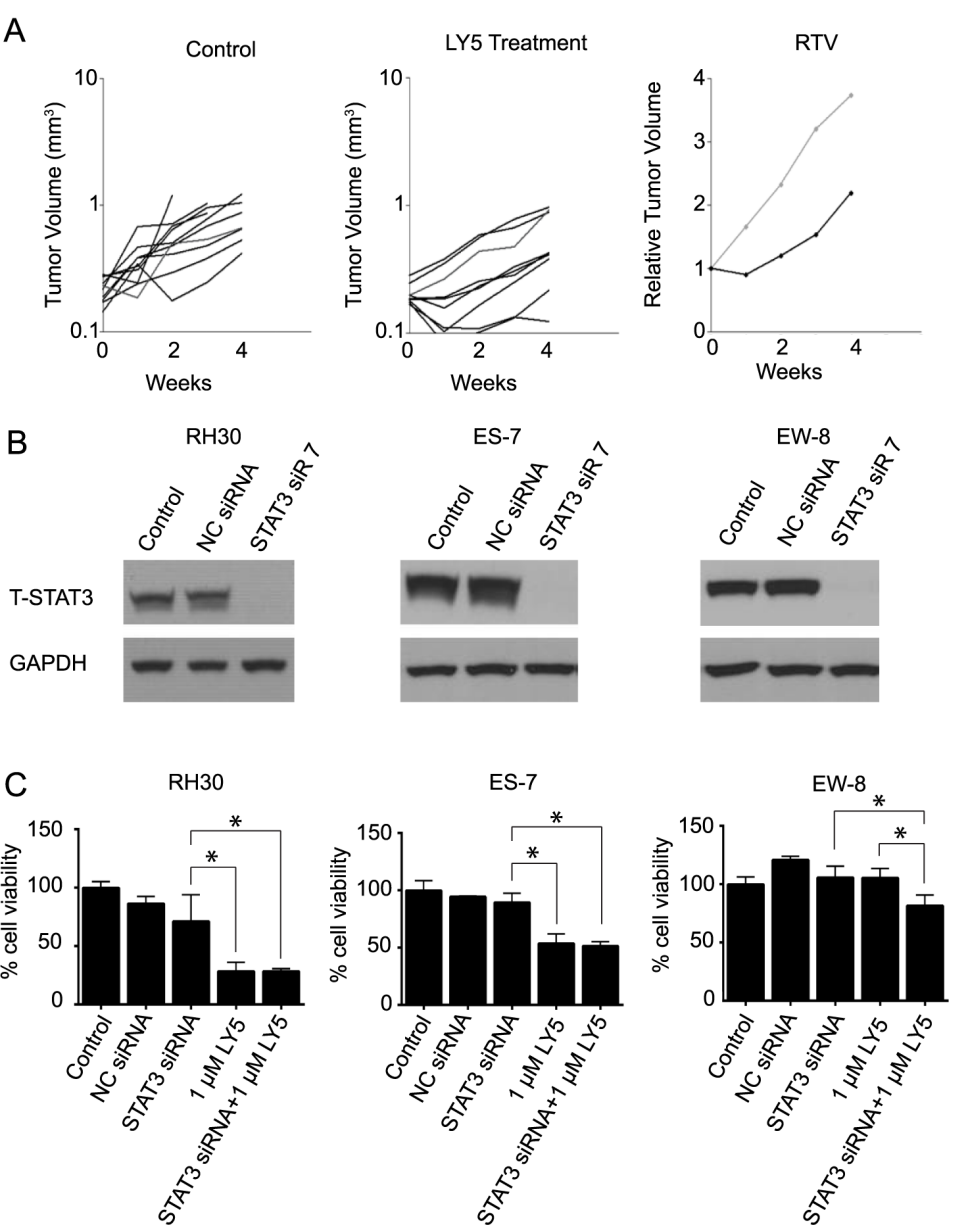
Effects of LY5 are not mediated via STAT3 inhibition. (A) Antitumor activity of LY5 against OS-1 PDX model of OS. Left panel, growth of individual control (untreated OS-1 xenografts); Center panel, growth of individual OS-1 xenografts in mice receiving LY5 (40 mg/kg twice daily for 5 days/week for 4 consecutive weeks); Right panel, median relative tumor growth (RTV) from control and treated groups. (B) Sarcoma cell lines (RMS cell line RH30 and ES cell lines ES-3 and ES-7) were transfected with either negative control siRNA (NC siRNA) or STAT3-targeting siRNA #7. After 72 hours of transfection, cells were harvested and analyzed for total (T) STAT3 and GAPDH protein expression by immunoblotting. (C) RH30, ES-7, and EW-8 cell lines were transfected with either NC siRNA or STAT3-targeting siRNA. After 48 hrs, cells were incubated with LY5 (1μM) for an additional 48 hrs. Cell viability was then determined using Alamar Blue staining. Data represent the mean ± standard deviation, n = 3. * denotes p < 0.05 by ANOVA.

To further examine the efficacy of LY5, we administered LY5 using subcutaneous PDX models of RMS (RH10 and RH36) and OS (OS-17 and OS-1). RH10, RH36 and OS-1 were derived from tissue explants, therefore no corresponding cell lines exist. Tumors were harvested 2 hrs after final drug administration. Similar to the lung metastasis growth in Fig 4C, LY5 did not decrease PDX growth compared to control (Table 1, Fig 6A). In addition, STAT3 phosphorylation was not observed in control or treated tumor derived from OS-17, and inhibition of STAT3 phosphorylation was not observed in OS-1 PDX models treated with 40 mg/kg twice daily.

**Table 1:** LY5 treatment did not inhibit RMS or OS xenograft tumor growth.

Tumor Line	Treatment	Survival (%)	Median day to event	Log rank p-value	Event-free survival (Treatment/Control)	Group Response
Rh10	Control	100	16.8			
	10 mg/kg^a^	100	18.7	0.1432	1.11	PD1^e^
OS-17^b^	Control	100	17			
	10 mg/kg^b^	90	19.7	0.8852	1.15	PD1
Rh36^b^	Control	100	17.4			
	10 mg/kg^b^	100	16.5	0.7448	0.94	PD1
OS-1^c^	Control	100	NE			
	40 mg/kg^c^	90	NE	0.1065	NE^d^	PD2^f^

^a^Treated daily for five days for four consecutive weeks

^b^Treated daily for 28 days

^c^Treated twice per day for five days for four consecutive weeks

^d^NE, Not evaluable

^e^PD1, Progressive disease 1, <50% tumor growth inhibition (TGI)

^f^PD2, Progressive disease 2, >50% TGI, >25% tumor progression

To address whether LY5 might have anticancer effects independent of STAT3, we transfected RMS, ES, and OS cells with siRNA targeting STAT3 (Fig 6B and S2A Fig) and then treated cells with increasing concentrations of LY5 (Fig 6C and S2B Fig). For STAT3 knockdown (>90%) was confirmed in all cell lines by western blot analysis. Compared to the control siRNA or untreated cells, knockdown of STAT3 had only a modest effect in reducing proliferation. The effect of 1 μM LY5 treatment was significantly different from STAT3 siRNA (p < 0.05) in RH30 and ES-7 cell lines. Importantly, the response to LY5 was comparable whether or not STAT3 had been depleted, indicating that its anti-proliferative activity was independent of STAT3. These data strongly suggest that the effect of LY5 on cell viability was independent of STAT3, supporting the notion that an off-target effect is responsible for the anti-proliferative effects of LY5.

## Discussion

STAT3 is a transcription factor involved in cytokine and receptor kinase signal transduction and its activation in tumor cells promotes metastasis and resistance to chemotherapy. LY5 was developed as a small molecule allosteric inhibitor of STAT3 building off of a prior analog LLL12 that had good activity *in vitro* but poor PK properties. Previous studies had described LY5 to have potential anti-tumor activity [17–21] but its true activity against sarcomas both *in vitro* and *in vivo* had not yet been characterized. The present study was performed to generate preclinical data in potential support of the continued development of LY5 as a novel cancer therapeutic. Our data showed that LY5 has excellent target specificity for STAT3, anti-proliferative activity at nanomolar drug concentrations against several sarcoma cell lines, and promising oral bioavailability in mice and dogs. However, drug concentrations needed to achieve tumor growth inhibition in both *ex vivo* and *in vivo* assays were discordant with those necessary to achieve target inhibition *in vitro*, suggesting that the anti-tumor effects of LY5 are not due to inhibition of STAT3 phosphorylation. The mechanism responsible for this observed discrepancy is currently unknown, however our results indicated that the anti-cancer activity observed in sarcoma cells exposed to LY5 was likely due to an off-target effect(s). Because LY5 demonstrated a lack of *in vivo* activity further investigation of these potential mechanisms is currently being pursued.

Experimental evidence has demonstrated that inhibition of JAK/STAT3 signaling impairs proliferation and survival in sarcoma cell lines, supporting the notion that STAT3 is a relevant therapeutic target [2,33–35]. In contrast, almost complete knockdown of STAT3 in the sarcoma cell lines used in this study failed to decrease proliferation in any cell line except RH30. These data indicate that the relationship between STAT3 and sarcoma cell survival is complicated and significant discrepancies in pathway relevance may be observed when studies are performed *in vitro* versus *in vivo*. Indeed, our unpublished data suggest that STAT3 may be more important for tumor cell-microenvironment interactions rather than for direct cell survival.

Clinical application of STAT3 inhibitors has been challenging. While a variety of small molecule and nucleic acid antisense inhibitors have been evaluated in the preclinical setting and in early-phase clinical trials, none have yet attained FDA approval. As previously discussed, we had developed another small molecule inhibitor of STAT3 (LLL12) that reduced proliferation, and induced apoptosis and cell cycle arrest of a variety of tumor cell lines [15,16,36]. However, this was found to have poor oral bioavailability and unfavorable PK. Therefore, LY5 was created by utilizing the fragment from LLL12 containing napthoquinonesulfonamide (thought to provide most of the binding energy to STAT3), and combining it with a pyridine ring to provide binding specificity to the SH2 domain side pocket of STAT3. The resultant compound, LY5, was designed as an allosteric inhibitor of STAT3 with proposed increased target specificity, oral bioavailability and ease of large-scale synthesis. Our data demonstrated that LY5 was indeed specific for STAT3, exhibiting no effects on other STAT family members. However, its lack of biologic activity *in vivo* combined with data generated from siRNA studies downregulating STAT3 expression in sarcoma cell lines strongly suggested that its effects *in vitro* were not tied to inhibition of STAT3 phosphorylation.

While investment in pharmaceutical research and development has increased substantially in the past two decades, productivity has declined [37]. One of the key technical factors contributing to project failures is lack of efficacy secondary to working on the wrong target for the disease of interest [38]. Another struggle is the language of failure, where the association of a project with ‘failure’ leads organizations to discard negative project related data rather than report the results [39]. Although some STAT3 inhibitors have been identified to induce antitumor effects *in vitro* and *in vivo*, few STAT3 inhibitors have been thoroughly studied for *in vivo* efficacy and pharmacology [40]. Since PK screening using various dose regimens and administration routes increases likelihood of success in preclinical testing [41], we examined the plasma bioavailability of LY5 with IV, IP, and oral delivery in mice and dogs, with the intention of evaluating drug activity in a spontaneous large animal model of cancer, canine OS. Despite the favorable PK profile of LY5 in both mice and dogs, the decision to discontinue development of this drug was made based on the lack of consistent *in vivo* activity that could be tied to a defined PK/PD relationship. Reporting results such as these may help combat the language of failure and improve outflow from the drug pharmaceutical pipeline.

## Conclusions

The novel small molecule inhibitor LY5, inhibited STAT3 phosphorylation and tumor cell viability *in vitro* and exhibited favorable PK properties in both mice and dogs including good oral bioavailability. However, the concentrations of LY5 necessary to observe an anti-tumor effect *in vivo* were discordant with the concentrations of LY5 necessary for inhibition of STAT3 phosphorylation. Further, LY5 anti-proliferative activity was similar in control cells and cells where STAT3 had been depleted, indicating that the anti-tumor effects of LY5 are not due to STAT3 inhibition.

## Supporting information

S1 Fig Kinomescan results for LY5. LY5 exhibits no interactions sterically or allosterically to the active sites of 96 protein or lipid kinases at 1 μM LY5 (A), mapped S-Score (35) = 0. At 5 μM LY5 (B), only interaction with PLK3 was identified, mapped S-score (35) = 0.01.
(PDF)

S2 Fig LY5 does not enhance the effects on cell viability in cells without STAT3 expression. (A) ES cell line ES-3 was transfected with either negative control siRNA (NC siRNA) or STAT3-targeting siRNA #7. After 72 hrs of transfection, cells were harvested and analyzed for total (T) STAT3 and GAPDH protein expression by immunoblotting. (B) RH30, ES-3, ES-7, and EW-8 cells were transfected with NC siRNA or siRNA targeting STAT3 for 48 hrs, followed by incubation with 0.5 μM or 1 μM LY5 for another 48 hrs. Cell viability was evaluated using Alamar Blue staining. Samples were set up as triplicates.
(PDF)

S1 Table Non-compartmental PK parameters. Comparison of maximum drug concentration (Cmax), time at Cmax (Tmax) drug exposure (AUC), and oral bioavailability (F) of LY5 in mice (n = 10 for IV, PO, and IP routes) (A) and two study dogs (B and C).
(PDF)
