# Supplementary figures and images for: Target specificity, in vivo pharmacokinetics, and efficacy of the putative STAT3 inhibitor LY5 in osteosarcoma, Ewing's sarcoma, and rhabdomyosarcoma
Source: PLoS One. 2017 Jul 27;12(7):e0181885. doi: 10.1371/journal.pone.0181885 (PMC5531494; doi:10.1371/journal.pone.0181885)

S1 Fig

A

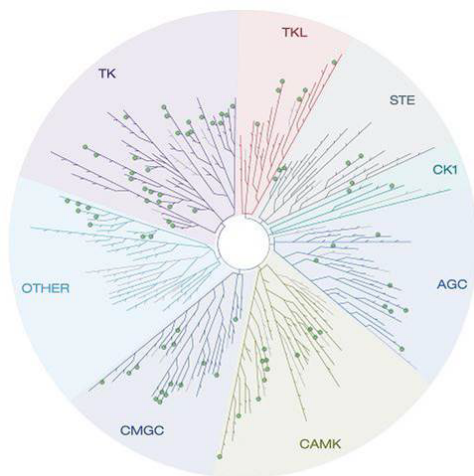

B

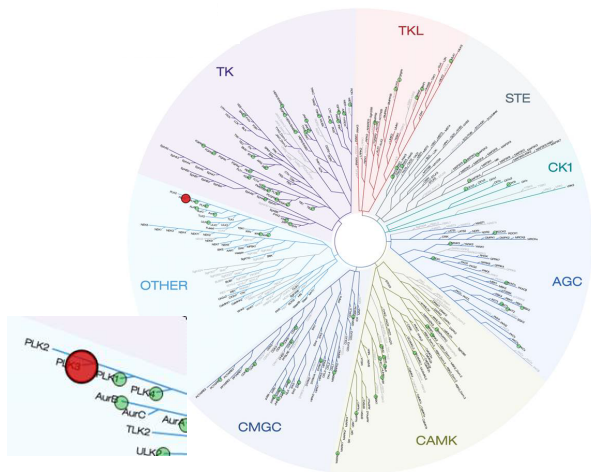

Supplement: S1 Fig — LY5 exhibits no interactions sterically or allosterically to the active sites of 96 protein or lipid kinases at 1 μM LY5 (A), mapped S-Score (35) = 0. At 5 μM LY5 (B), only interaction with PLK3 was identified, mapped S-score (35) = 0.01. (PDF) [file pone.0181885.s001.pdf]

## S2 Fig

A

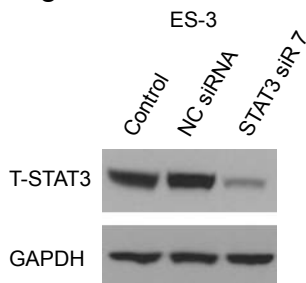

B

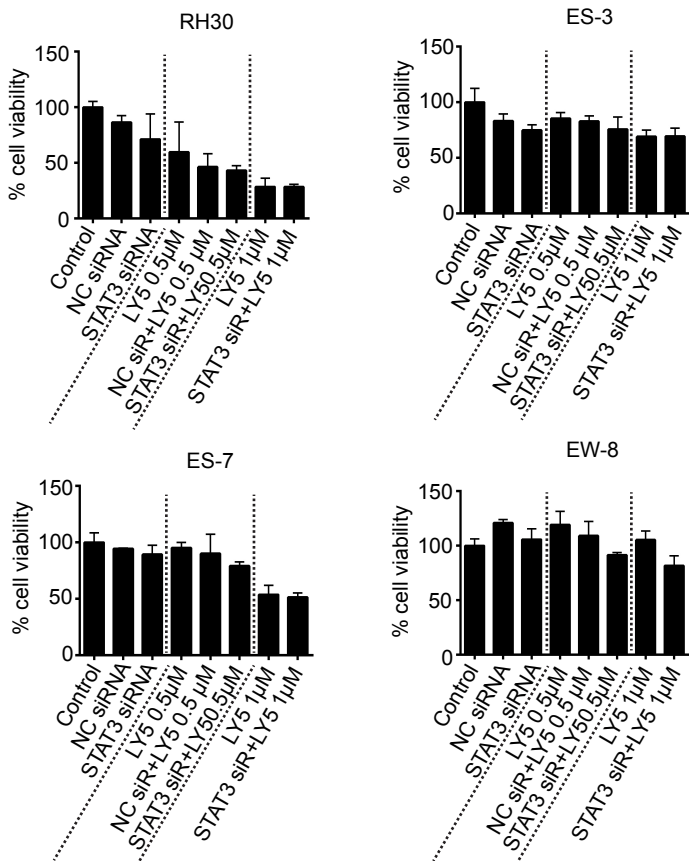

Supplement: S2 Fig — (A) ES cell line ES-3 was transfected with either negative control siRNA (NC siRNA) or STAT3-targeting siRNA #7. After 72 hrs of transfection, cells were harvested and analyzed for total (T) STAT3 and GAPDH protein expression by immunoblotting. (B) RH30, ES-3, ES-7, and EW-8 cells were transfected with NC siRNA or siRNA targeting STAT3 for 48 hrs, followed by incubation with 0.5 μM or 1 μM LY5 for another 48 hrs. Cell viability was evaluated using Alamar Blue staining. Samples were set up as triplicates. (PDF) [file pone.0181885.s002.pdf]
