# Supplementary material for: Target specificity, in vivo pharmacokinetics, and efficacy of the putative STAT3 inhibitor LY5 in osteosarcoma, Ewing's sarcoma, and rhabdomyosarcoma
Source: PLoS One. 2017 Jul 27;12(7):e0181885. doi: 10.1371/journal.pone.0181885 (PMC5531494; doi:10.1371/journal.pone.0181885)
Supplement: S1 Table — Comparison of maximum drug concentration (Cmax), time at Cmax (Tmax) drug exposure (AUC), and oral bioavailability (F) of LY5 in mice (n = 10 for IV, PO, and IP routes) (A) and two study dogs (B and C). (PDF) [file pone.0181885.s003.pdf]

S1 Table

A

PK parameters	IV	PO	IP
Tmax (hr)	0.083	0.5	0.083
Cmax (nM)	1662	509	545
AUC all (nM*hr)	1138	784	717
AUC inf-obs (nM*hr)	1142	898	794
F (%)	100	78.6	69.5

B

Animal ID/Route→	Dog #1			Dog #2		
	IV	PO fed	PO w/ fast	IV	PO fed	PO w/ fast
Dosage (mg/kg)	0.97	1.28	0.9	0.97	1.28	0.9
Cmax (nM)	5586	206	1284	3222	60.4*	500
AUC (nM*hr)	5096	924	2438	3917	258*	1611
F (%)	100	13.7	51.6	100	5*	44.3

*dog vomited 10 min after dosing

C

PK Parameters↓	Dog #1		Dog #2	
Dosage →	25 mg	50mg	25 mg	50mg
AUC last (hr*nM)	2623	2922	2506	5929
AUC inf-obs (hr*nM)	2688	2970	2530	5956
Tmax (hr)	2	3	2	4
Cmax (nM)	230	840	516	909
